# Apathy and Impulsivity Co‐Occur in Huntington's Disease

**DOI:** 10.1002/brb3.70061

**Published:** 2024-09-30

**Authors:** Lee‐Anne Morris, Kyla‐Louise Horne, Laura Paermentier, Christina M. Buchanan, Michael MacAskill, Daniel Myall, Masud Husain, Richard Roxburgh, Tim Anderson, Campbell Le Heron

**Affiliations:** ^1^ Department of Medicine University of Otago Christchurch New Zealand; ^2^ New Zealand Brain Research Institute Christchurch New Zealand; ^3^ Department of Neurology Auckland City Hospital, Te Whatu Ora Health Auckland New Zealand; ^4^ Centre for Brain Research Neurogenetics Research Clinic University of Auckland Auckland New Zealand; ^5^ Department of Experimental Psychology University of Oxford Oxford UK; ^6^ Nuffield Department of Clinical Neurosciences University of Oxford Oxford UK; ^7^ Department of Neurology Christchurch Hospital, Te Whatu Ora Health Christchurch New Zealand

**Keywords:** apathy | Huntington's disease | impulsivity | quality of life

## Abstract

**Background:**

Apathy is a debilitating behavioral change in Huntington's disease (HD), but impulsivity in HD has not been well documented, and the co‐occurrence of these behaviors in HD has not been investigated.

**Objective:**

Our objective was to determine whether apathy and impulsivity co‐occur in people with HD and their associations with quality of life.

**Methods:**

Carriers of Huntington's gene expansion (premanifest to mild motor manifest disease; *n *= 42) along with healthy controls (*n *= 20) completed measures of apathy (Apathy Evaluation Scale and Apathy Motivation Index) and impulsivity (Barratt Impulsiveness Scale‐11 and UPPS‐P impulsivity scale), along with mood, cognition, clinical, and quality of life measures. Apathy and impulsivity measures were each reduced to a single metric per patient using principal component analysis. Correlations and multiple linear regression models determined associations between apathy and impulsivity and the potential influence of other covariates.

**Results:**

Apathy and impulsivity were significantly correlated (*r *= 0.6, *p *< 0.001, 95% CI [0.36, 0.76]) in HD, with this association remaining after controlling for depressive symptoms, motor disease severity, and cognitive function. Furthermore, apathy and depressive symptoms were associated with poorer quality of life.

**Conclusions:**

Apathy and impulsivity co‐occur in individuals with premanifest to mild manifest HD and have a significant impact on wellbeing. We add to a growing evidence body that apathy and impulsivity may be intrinsically linked.

## Graphical abstract

Morris et al. investigated the co‐occurrence of apathy and impulsivity in Huntington's disease. These behavioral changes were significantly associated, and this association was not otherwise explained by cognition, mood, or motor disease severity. This suggests a common neurobiological mechanism underpinning these seemingly disparate behaviors—an important treatment target given their impact on wellbeing and life quality.

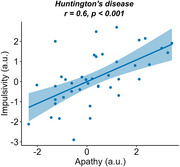

## 1 Introduction

Although traditionally considered representing opposite ends of a spectrum of behavioral disturbance (Kirschner et al. 2020), the co‐occurrence of apathy and impulsivity is increasingly recognized. This is evident on trait level in healthy individuals (Petitet et al. 2021, 2022) and clinically in individuals with brain disorders, such as Parkinson's disease (Scott et al. 2020; Sinha, Manohar, and Husain 2013), frontotemporal lobar degeneration syndromes (Lansdall et al. 2017; Passamonti, Lansdall, and Rowe 2018), progressive supranuclear palsy (Kok et al. 2021), Alzheimer's disease (Zhao et al. 2016), schizophrenia (Velligan et al. 2002), and attention deficit hyperactivity disorder (Torrente et al. 2011). In these conditions, some people develop apathy and impulsivity in isolation, but in many, both are present. This raises the possibility of a common mechanism underlying trait‐level behaviors that, when disrupted in the setting of pathology, leads to clinically relevant behavioral changes.

Behavioral changes, along with motor and cognitive decline, are an intrinsic part of Huntington's disease (HD), a genetic neurodegenerative disorder that lies at the intersection between neurology and psychiatry. HD is rare, with a prevalence of 10.6–13.7 per 100,000 in Western populations, onset typically between ages 30 and 50, and disease progression over approximately two decades (Tabrizi et al. 2013). In particular, apathy is highly prevalent and worsens with disease progression (Tabrizi et al. 2013; Thompson et al. 2012). Apathy is routinely evaluated in HD, for example, as part of the short Problem Behaviors Assessment used in an international HD study test battery (Sathe et al. 2021). In addition, studies designed to probe the underlying cognitive mechanisms of apathy in HD, by using specially designed behavioral tasks, are emerging (McLauchlan et al. 2019; Nair et al. 2021). Impulsivity, in contrast, has not been well characterized in HD and is not routinely included in HD studies. Anecdotally, clinicians treating people with HD report impulsivity is common, but it is unclear whether these people are also apathetic. In light of evidence that apathy and impulsivity commonly co‐occur in general, it would be reasonable to *assume* that apathy and impulsivity co‐occur in HD too. However, it is imperative to *empirically* ascertain whether, in fact, apathy and impulsivity do co‐occur in HD or whether the development of apathy in HD occurs without concomitant increases in impulsiveness. There are a number of reasons why this is important. First, there is a dire need for apathy treatments in HD. Should impulsivity be intrinsically linked to apathy in HD, then treatments and clinical trial endpoints may need to account for this by including baseline assessments and clinical endpoints for *both* apathy and impulsivity. Second, the neuropathological course in HD is increasingly well described, with loss of GABAergic medium spiny neurons in the dorsal striatum as one of the earliest structural changes in the brain (Tabrizi et al. 2022). Understanding at a descriptive level the relationships between behavioral disruptions can pave the way for more hypothesis‐driven work to bridge the gap between brain pathology—particularly within networks relevant to motivated behavior—and clinical phenotypes. Finally, it may be necessary to include impulsivity assessments as part of larger HD study batteries, but evidence for this is required.

Both apathy and impulsivity are currently conceptualized as multidimensional constructs, with differing expressions across individuals and environments. Apathy, broadly defined as *loss of motivation leading to an observable reduction in goal‐directed behavior*, has been proposed to occur along emotional, social, cognitive, and behavioral dimensions (Levy and Dubois 2006; Marin, Biedrzycki, and Firinciogullari 1991), although this conceptualization has recently been questioned (Dickson and Husain 2022). Indeed, items from apathy scales do not load onto factors suggestive of these dimensions (Dickson and Husain 2022). Impulsivity is construed as having motoric forms (encompassing premature responding and reduced ability to inhibit actions) and decisional forms (preference for immediately available rewards, lack of appropriate forethought leading to rapid decisions, and increased risk‐seeking behavior) (Dalley and Robbins 2017). In HD, neuroimaging studies of apathetic and impulsive traits in isolation have identified neural correlates in similar brain regions, including the mid and dorsal anterior cingulate cortex and ventral striatal regions, among others (De Paepe et al. 2021; Gray et al. 2013; Martínez‐Horta et al. 2018; Rao et al. 2014; van den Bogaard et al. 2011). These observations provide some evidence for shared or overlapping anatomical substrates, whereas a recent review has proposed that disruption of specific components of reward and cost evaluation (value‐based decision‐making) provides a common framework within which to understand the mechanisms underlying both apathy and impulsivity (Morris et al. 2022). However, before exploring such possibilities further, there is a crucial need to understand the basic relationship between these behavioral disturbances in individual people with HD.

In the current work, we investigated whether self‐reported apathy and impulsivity co‐occurred in HD. Having a study partner was not a requirement for this study, and many participants attended sessions alone. Behavioral scales were self‐reported, and thus, it is important to note that lack of insight or awareness (anosognosia) may lead to underreporting of some behavioral symptoms in HD (Isaacs et al. 2020). Given our primary interest in the relationship between apathy and impulsivity in HD at the broadest level, we used global apathy and impulsivity scores in our primary analyses in this work. As an additional analysis, we also examined the relationship between dimensions (as currently conceptualized) of each. Although our aim was not to compare apathy and impulsivity in HD against healthy controls, we included a cohort of healthy controls in the study to provide a comparison of self‐report scores from a non‐diseased setting. We hypothesized that self‐reported apathy and impulsivity would be positively correlated in HD. In addition, we predicted their presence would be associated with poorer quality of life.

## 2 Materials and Methods

### 2.1 Ethics

The study was approved by the Health and Disability Ethics Committee of the New Zealand Ministry of Health (21/CEN/242). Written consent was obtained from all participants, and research was conducted in accordance with the Declaration of Helsinki.

### 2.2 Participants

People with confirmed expansion of Huntington's gene (premanifest to mild motor manifest disease^1^) were recruited from two specialist HD clinics (Auckland and Christchurch, New Zealand, *n *= 42). The majority of participants were part of Enroll‐HD, a large international prospective longitudinal HD study (Sathe et al. 2021). Age‐ and gender‐matched healthy controls (*n *= 20) were recruited via local databases. Demographic and clinical data are presented in Table 1.

**TABLE 1:** Demographic and clinical characteristics of participants.

	Controls	HD	Test statistic
Participants	*n* = 20	*n *= 42	
Age	51 (18)	52 (14)	*t*(30.6) = −0.2, *p* = 0.8
Male: female (%male)	8:12 (40%)	20:22 (48%)	*χ* ^2^ (38, *n* = 62) = 37.4, *p* = 0.5
Education in years	15 (2.7)	14 (3.4)	*t*(45.1) = 1.6, *p* = 0.1
UHDRS total motor score /124	NA	13.8 (12.6)	NA
CAG repeat length	NA	42 (2)	NA
Antidepressant use, *n* (%)	4 (20%)	13 (31%)	*χ* ^2^ (1, *n* = 62) = 0.36, *p* = 0.5
Antipsychotic use, *n* (%)	0 (0%)	10 (24%)	*χ* ^2^ (1, *n* = 62) = 4.1, *p* = 0.04

*Note*: Table showing mean (standard deviation).

Abbreviation: UHDRS = Unified Huntington's Disease Rating Scale.

### 2.3 Disease, Cognitive, and Questionnaire Measures

Apathy was measured using the self‐rated Apathy Evaluation Scale (AES—self) (Marin, Biedrzycki, and Firinciogullari 1991), one of the most widely used apathy scales across different disorders, including HD (Clarke et al. 2007) and previously shown to correlate highly with caregiver‐rated apathy in HD (Mason and Barker 2015); and the self‐rated Apathy Motivation Index (AMI), recently developed to assess different putative dimensions of apathy (Ang et al. 2017). Impulsivity was measured using the self‐rated UPPS‐P (59‐item version), which evaluates 5 dimensions of impulsivity: positive urgency, negative urgency, lack of premeditation, lack of perseverance, and sensation seeking (Whiteside et al. 2005). In addition, participants completed the self‐rated Barratt Impulsiveness Scale‐11 (BIS‐11) (Patton, Stanford, and Barratt 1995). Depressive symptoms were measured using the self‐rated Beck Depression Inventory II (BDI‐II) (Beck, Steer, and Brown 1996). Because some elements of the BDI‐II probe motivational changes that overlap with apathy, a subscore, excluding these items, was calculated (dysphoria‐subscale), using the previously validated methodology (Kirsch‐Darrow et al. 2011). Cognition was screened using the Montreal Cognitive Assessment (MoCA) (Gluhm et al. 2013; Nasreddine et al. 2005; Videnovic et al. 2010). Fifteen participants had no MoCA score but did have a Mini‐Mental State Examination score (MMSE), which was converted to an MoCA score using a standardized conversion (van Steenoven et al. 2014). The WHO‐5 Well‐being Index was used to assess general wellbeing (Topp et al. 2015). Quality of life was assessed using a Cantril Ladder, on which participants rate their current life satisfaction on a scale of 1–10 (Cantril 1965). Motor disease severity was measured using the Unified Huntington's Disease Rating Scale–Total Motor Score (UHDRS‐TMS) section by an experienced neurologist (TA, RR, or CLH) (Huntington Study Group 1996). Central nervous system medication use was grouped according to drug class (antipsychotics, encompassing both typical and atypical; antidepressants, encompassing SSRIs, SNRIs, and TCAs), coded as either use *yes* or *no*, and utilized as binary variables in this study.

### 2.4 Statistical Analyses

Statistical analyses were performed using R (R Core Team 2022). Group differences were tested using Welch's *t*‐tests or chi‐square tests, depending on the variables. Correlations were tested using Pearson's correlation coefficient, as variables were normally distributed. Effect sizes were calculated using Cohen's d. Each participant completed at least one measure for apathy and at least one for impulsivity, respectively. Missing data were as follows: Overall, 3 people were missing AMI scores; 2, AES scores; 5 were missing UPPS‐P scores, 13 BIS‐11 scores, and 5 were missing BDI‐II scores. Missing data were imputed using *mice* in R (van Buuren and Groothuis‐Oudshoorn 2011). For each scale, the mean of five imputed scores generated by *mice* per individual was used as input to the principal component analyses, where scale scores were missing.

Rather than analyze multiple correlated instruments and to reduce the impact of missing data, we used principal component analyses (*FactoMineR* in R) to obtain one metric for apathy (AES and AMI scores), and one for impulsivity (BIS‐11 and UPPS‐P scores) per individual. This is in line with previous approaches (Lansdall et al. 2017; Petitet et al. 2022). Each single score represented their expression of the first principal component (which captured most of the variance of each construct's two questionnaires). These components, respectively, accounted for 88% of the variance in apathy measures and 74% of the variance in impulsivity measures. Multiple regression models were then performed using *lm* from base R, using the PCA‐derived apathy and impulsivity scores. Formal model comparison was performed using covariates dysphoria (BDI‐II dysphoria), motor disease score (UHDRS‐TMS), global cognitive function (MoCA), age, and sex to further investigate whether alternative factors might explain any observed relationship between apathy and impulsivity. All variables were *z*‐scored.

Finally, to investigate associations between putative dimensions of apathy and impulsivity, Pearson's correlations were performed between subscales of the AMI and UPPS‐P, both of which have previously undergone formal factor analysis and validation (Ang et al. 2017; Whiteside et al. 2005; Whiteside and Lynam 2001). Correlations were corrected for multiple comparisons using the *false discovery rate* method (Benjamini and Hochberg 1995).

Given that the use of antipsychotic medication was significantly higher in people with HD compared to controls (*p *= 0.04), we reran all models with antipsychotic use (y/n) as an additional covariate. However, there was no evidence of associations with this variable and apathy, impulsivity, or quality of life/wellbeing in our cohort, and its inclusion did not improve model fit, so we report models run without this covariate.

## 3 Results

Compared to healthy controls, people with premanifest to mild motor manifest HD had higher levels of apathetic (AES and AMI) and impulsive traits (BIS‐11 but not UPPS‐P) (AES mean difference = 6.6, *t*(_58_) = −3.6, *p *= 0.0006, *d *= 0.88; AMI mean difference = 5.9, *t*(_51_) = −2.9, *p *= 0.006, *d *= 0.75; BIS‐11 mean difference = 7.2, *t*(_42_) = −2.2, *p *= 0.03, *d *= 0.64; UPPS‐P mean difference = 9, *t*(_47_) = −1.3, *p *= 0.2, *d *= 0.34). There were no significant group‐level differences between controls and HD with regards to quality of life or global cognition (MoCA). Although people with HD had numerically higher overall depressive symptoms than controls, as well as higher dysphoria scores, these did not reach statistical significance (Table 2).

**TABLE 2:** Behavioral and cognitive characteristics of participants.

	Controls	HD	Test statistic
Participants	*n* = 20	*n* = 42	
**Apathy**			
AES‐s /72	24.6 (4.4)	31.2 (9.6)	*t*(57.8) = −3.6, *p* = 0.0006
Behavioral	6 (1)	8 (3)	
Cognitive	11 (2)	14 (5)	
Emotional	3 (1)	4 (1)	
AMI /72	17.8 (6.5)	23.7 (9.2)	*t*(51.1) = −2.9, *p* = 0.006
Behavioral	6 (3)	9 (5)	
Social	7 (4)	9 (5)	
Emotional	5 (3)	6 (4)	
**Impulsivity**			
BIS‐11 /120	53.5 (8.9)	60.7 (13.3)	*t*(41.9) = −2.2, *p* = 0.03
Attentional	14 (3)	16 (4)	
Non‐planning	20 (4)	23 (6)	
Motor	20 (3)	22 (4)	
UPPS‐P /236	115 (23.7)	124 (30)	*t*(47.2) = −1.3, *p* = 0.2
Lack of perseverance	17 (5)	21 (6)	
Lack of premeditation	21 (6)	22 (5)	
Urgency (positive)	21 (8)	26 (11)	
Urgency (negative)	24 (8)	26 (9)	
Sensation seeking	32 (8)	30 (8)	
**Depressive symptoms**			
BDI‐II /63	6.7 (5.4)	9.9 (9.8)	*t*(55.0) = −1.6, *p* = 0.1
BDI dysphoria /33	3 (3.3)	4.7 (6.1)	*t*(55.0) = −1.4, p = 0.2
**Quality of life**			
WHO‐5 /100	66.2 (18.4)	68.6 (17)	*t*(36.1) = −0.5, *p* = 0.6
Cantril Ladder /10	7.6 (1.3)	7.7 (1.6)	*t*(46.2) = −0.4, *p* = 0.7
**Cognition**			
MoCA /30	27.8 (1.9)	26.2 (4.7)	*t*(59.1) = 1.9, *p* = 0.07

*Note*: This table is showing mean (standard deviation). AES‐s = Apathy Evaluation Scale (self‐report); AMI = Apathy Motivation Index; BIS‐II = Barratt Impulsiveness Scale—II; UPPS‐P = UPPS‐P Impulsiveness Scale; BDI‐II = Beck Depression Inventory—II; WHO‐5 = WHO Well‐being Index—5; MoCA = Montreal Cognitive Assessment.

### 3.1 Apathy and Impulsivity Co‐Occur Within Individuals With HD

**FIGURE 1:**
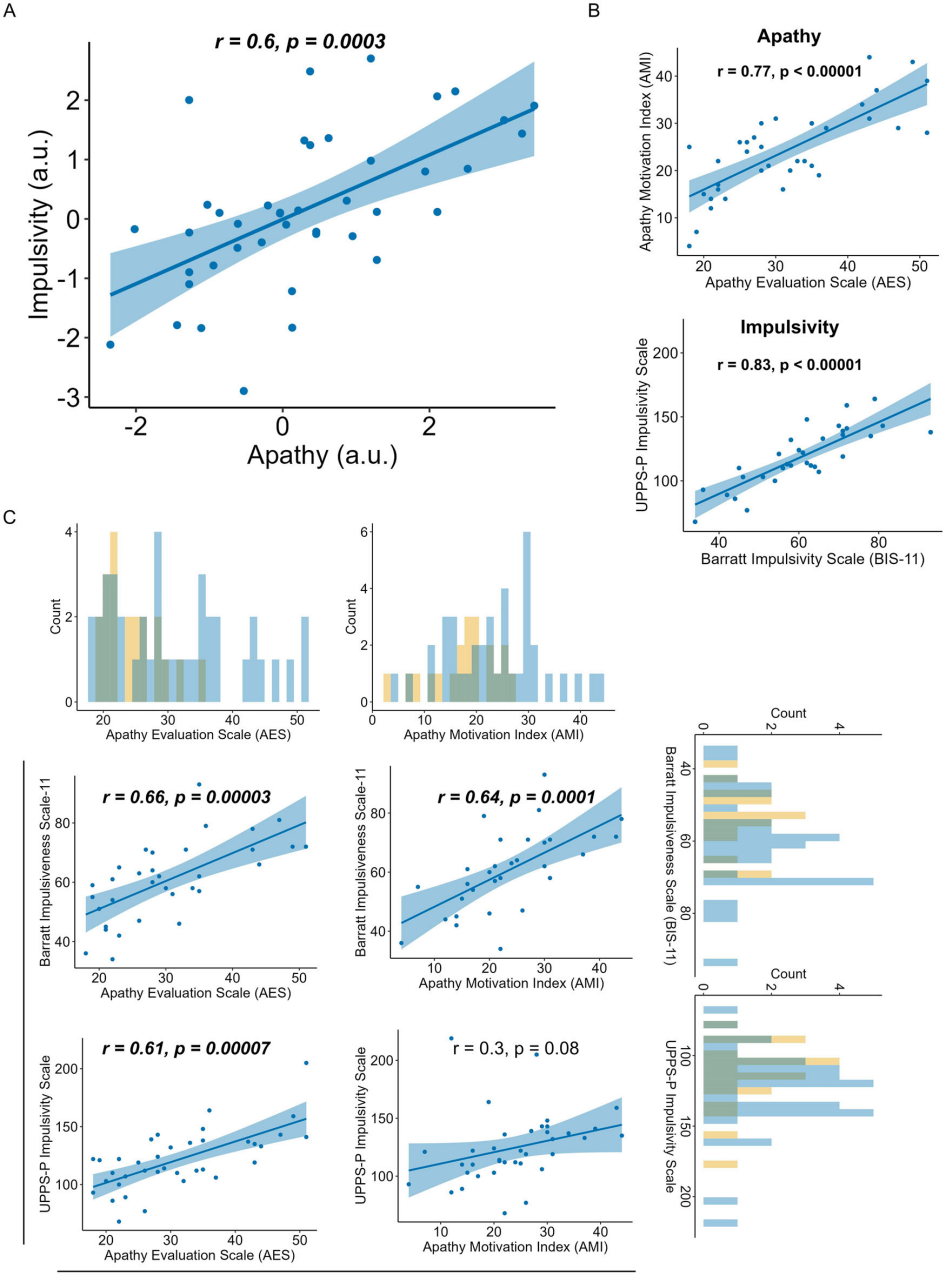
Correlation between apathy and impulsivity in Huntington's disease. (A) Apathy and impulsivity scores were positively correlated in people with Huntington's disease (Pearson's correlation, blue line). Scores were calculated from a principal component analysis of apathy (AMI and AES) and impulsivity (BIS‐11 and UPPS‐P) questionnaires. (B) Questionnaires assessing apathy and impulsivity, respectively, were strongly correlated within each construct in people with Huntington's disease. (C) Relationships between individual apathy and impulsivity questionnaire raw scores. Histograms depict frequency of raw scores for each measure. Pearson's correlations are shown for Huntington's disease. Control data are superimposed in the histograms to demonstrate the distribution of these measures in the normal population. a.u. = arbitrary units.

Apathy and impulsivity were significantly positively correlated in people with HD (*r *= 0.6, *p *< 0.001, Figure 1A). That is, greater levels of apathetic traits were associated with greater levels of impulsive ones. To further investigate the potential for confounders to be mediating this relationship, a formal model comparison between the simplest model, in which only impulsivity was included as a predictor of apathy, and extended models (including covariates dysphoria, motor disease severity, global cognition, age, and sex) was performed. The winning model (based on both AIC and BIC scores) contained only impulsivity (impulsivity: *β *= 0.8, *t *= 4.7, *p *< 0.0001; Table S1).

Comparison of individual questionnaires (AES, AMI, BIS‐11, and UPPS‐P) in people with HD (rather than PCA output) demonstrated that the AES was significantly correlated with both impulsivity measures. The AMI was significantly correlated with the BIS‐11 but not the UPPS‐P (Figure 1C). Finally, within individuals with HD, total apathy scores from the AES and AMI were strongly correlated (*r *= 0.77, *p *< 0.000001), as were total impulsivity scores from the BIS‐11 and UPPS‐P (*r *= 0.83, *p *< 0.000001; Figure 1B).

### 3.2 Apathy and Impulsivity Traits Within the Healthy Control Population

None of the controls scored above clinical cut‐offs for apathy or impulsivity. After controlling for dysphoria, there was a significant association between apathy and impulsivity in this healthy group (*β *= 0.6, *t *= 3.0, *p *= 0.008). Further details of the control group correlations are provided in Figure S1 and Table S3.

### 3.3 Apathy Subscales Show Differential Patterns of Association

**FIGURE 2:**
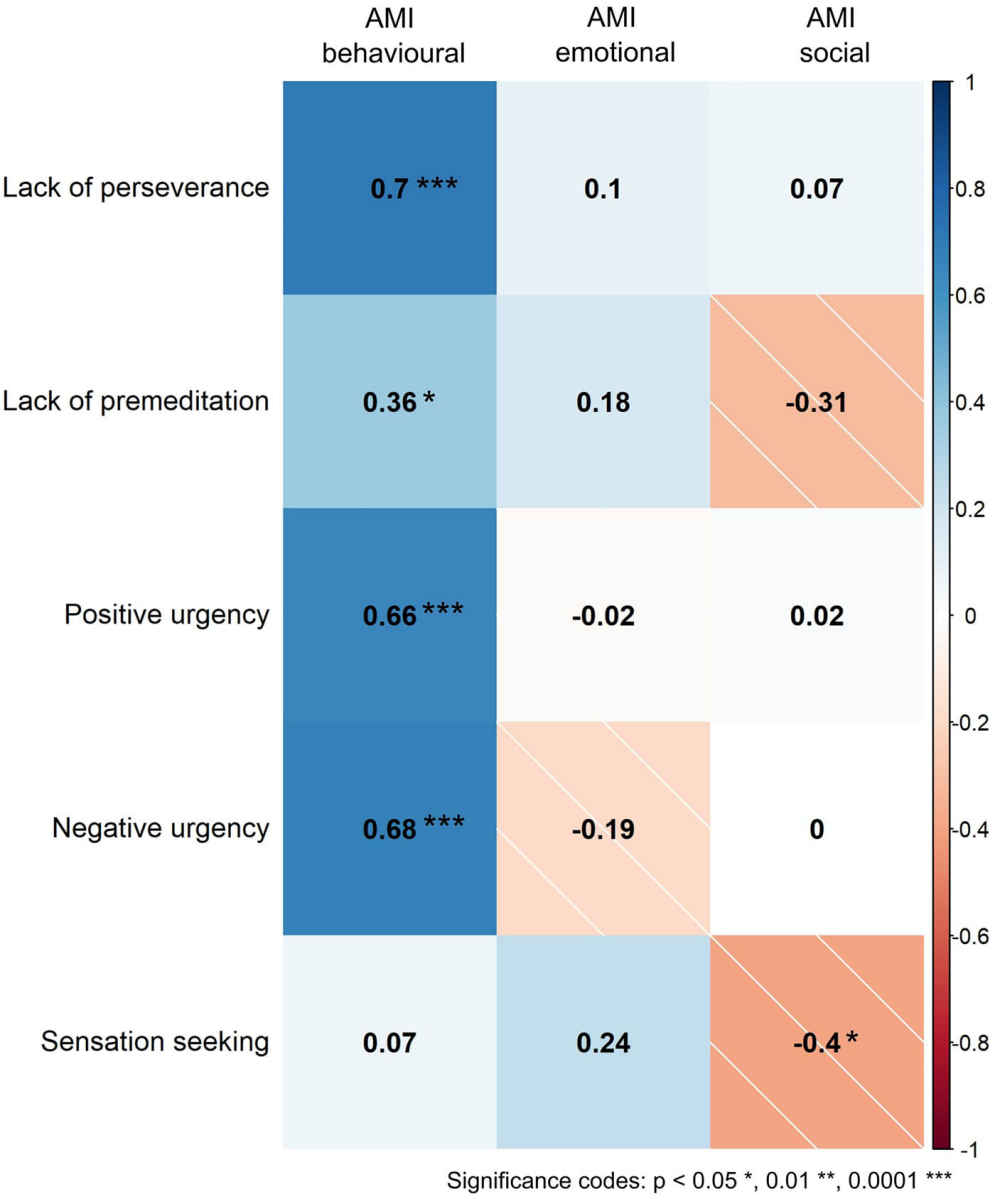
Subscales of apathy and impulsivity measures. Apathy (AMI) and impulsivity (UPPS‐P) subscale correlation coefficients for participants with Huntington's disease. Impulsivity traits lack of perseverance, positive urgency, and negative urgency showed significant correlations with behavioral apathy, after correcting for multiple comparisons. Social and emotional apathy were not significantly correlated with impulsivity dimensions (except for a negative correlation between social apathy and sensation seeking). Color intensity denotes strength of correlation (lighter = weaker, darker = stronger), and color palette denotes direction of correlation (blue = positive, red with hatching = negative).

Although total scores provide a global picture of apathetic or impulsive behavior, subscales potentially provide information about particular dimensions of these behaviors. In HD, statistically significant positive correlations between AMI behavioral apathy and the UPPS‐P impulsivity dimensions *lack of perseverance, positive urgency*, and *negative urgency* were evident. In contrast, there were no significant correlations between *emotional* or *social* apathy and any impulsivity dimensions (except for a negative correlation between *sensation seeking* and *social* apathy) (Figure 2). Correlation patterns between subscales in healthy controls are shown in Figure S2.

### 3.4 Apathy and Impulsivity Relationships With Quality of Life and Wellbeing in HD

**FIGURE 3:**
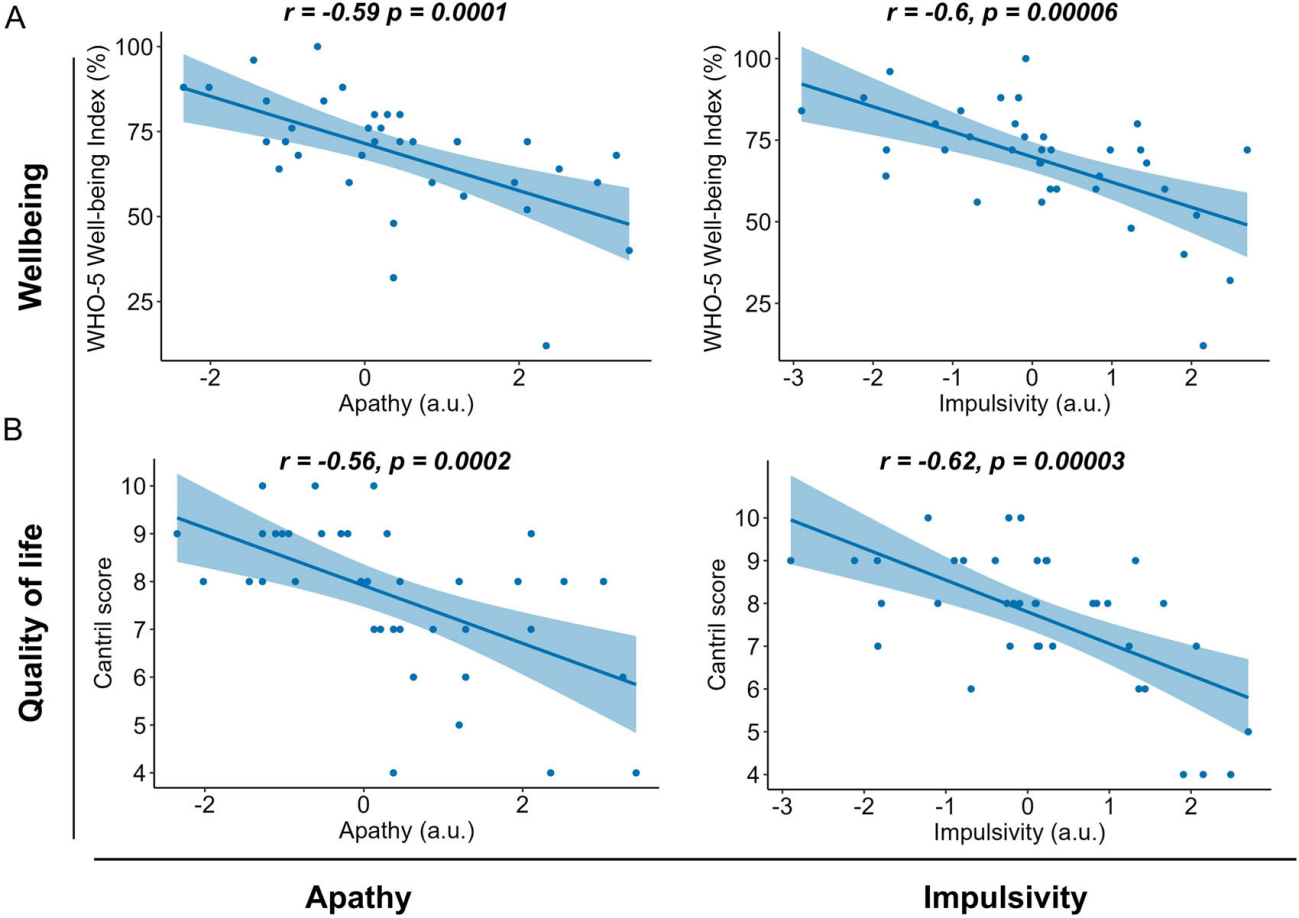
Quality of life and wellbeing correlations with apathy and impulsivity. In people with Huntington's disease, apathy and impulsivity were correlated with (A) decreased wellbeing and (B) poorer quality of life. After controlling for covariates in multiple regression models, apathy, but not impulsivity, remained a significant predictor of reduced quality of life in HD.

In HD, higher levels of apathy were significantly correlated with both lower wellbeing (WHO‐5 Well‐being Index score) and worse quality of life (Cantril Ladder) (WHO‐5: *r*(_40_) = −0.59, *p *= 0.0001; Cantril: *r*(_40_) = −0.56, *p *= 0.0002). Similarly, higher levels of impulsivity were significantly correlated with lower wellbeing and reduced quality of life (*r*(_40_) = −0.6, *p *= 0.00006); *r*(_40_) = −0.62, *p *= 0.00003; Figure 3). To investigate potential confounders of these correlations, we used multiple regression models to control for global cognition, dysphoria, motor disease severity, age, and sex. Apathy remained a significant predictor of wellbeing (*t *= −2.5, *p *= 0.01) and quality of life (*t *= −2.1, *p *= 0.04) after controlling for these other factors. In contrast, impulsivity was no longer significantly associated with either wellbeing or quality of life. Dysphoria was a significant predictor of wellbeing and quality of life in HD (Table 3). Healthy control associations are shown in Table S4 and Figure S3.

**TABLE 3:** Multiple regression model outputs showing predictors of wellbeing and quality of life in people with Huntington's disease.

	Wellbeing (WHO‐5 Well‐being Index)				Quality of life (Cantril Ladder)			
	Estimate	Std. Error	*t* value	*p* value	Estimate	Std. Error	*t* value	*p* value
**Apathy**	−6.1	2.5	−2.5	**0.02**	−0.5	0.3	−2.1	**0.04**
Impulsivity	0.9	2.7	0.3	0.73	−0.3	0.3	−1.0	0.31
**Depression**	−7.6	2.1	−3.7	**0.0009**	−0.4	0.2	−2.1	**0.048**
Motor disease severity	3.9	2.1	1.9	0.07	−0.3	0.2	−1.3	0.2
Cognition	−0.9	2.0	−0.5	0.65	−0.2	0.2	−1.1	0.26
Age	2.4	2.3	1.0	0.32	0.3	0.2	1.3	0.22
Sex: male	4.2	4.4	0.9	0.35	0.7	0.4	1.6	0.11

## 4 Discussion

Although there is now increasing evidence that apathy and impulsivity co‐occur in different clinical populations, their relationship in HD has been until now unexplored. In this study, we demonstrate that apathy and impulsivity are significantly associated within individuals with HD, across a spectrum of severity that includes clinically relevant levels of each, and that this relationship is not explained by other behavioral or mood‐related confounders. Importantly, these behavioral changes are associated with reduced wellbeing and poorer quality of life. Subscale analysis suggests that the overlap between these two behavioral phenotypes may occur between particular dimensions of each construct. Overall, these findings are consistent with recent theoretical proposals that the neural underpinnings of these seemingly disparate behavioral disturbances may be closely linked. They highlight the crucial need for understanding which processes have gone awry in apathy and impulsivity in HD, which in turn can inform the development of targeted treatment strategies.

Apathy and impulsivity were significantly positively associated in people with premanifest to mildly manifest HD. That is, people with higher levels of apathetic traits also had higher levels of impulsive behavioral traits. This association was not explained by potentially confounding factors, such as mood disorder, motor disability, or cognitive impairment. It also extended past the trait levels of each construct found in the healthy population, suggesting that when a person develops clinically significant apathy in the context of HD, they are also likely to be becoming more impulsive. To our knowledge, this is the first time the co‐occurrence of apathy and impulsivity has been demonstrated in HD. This association has, however, been shown in a growing number of other neurological and psychiatric disorders (Petitet et al. 2021; Sinha, Manohar, and Husain 2013; Torrente et al. 2011; Velligan et al. 2002; Zhao et al. 2016), and our study provides further convergent evidence for a close relationship between these two neurobehavioral disturbances. The importance of these changes is demonstrated by their relationship—even at early stages of HD—to quality of life and wellbeing, underscoring the need for careful screening for both. Although apathy is widely acknowledged as an important issue in HD and considered to be intrinsic to the disease process (Thompson et al. 2012), impulsivity has received less research and clinical attention and has not routinely been included in behavioral assessments, although this is changing (McDonell et al. 2020). At a clinical level, the results emphasize the importance of screening for features of each—particularly if either is identified, although the optimal measures to use for this in HD have not yet been clearly identified.

The co‐occurrence of impulsivity and apathy raises the clear possibility of shared cognitive mechanisms underpinning the development of phenotypically distinct problems. The demonstration of this association across many brain disorders points to a *trans‐diagnostic* viewpoint, whereby it is the disruption of specific cognitive processes—rather than the pathology causing the disruption—that is most important for their behavioral expression. Both apathy and impulsivity, at the broadest level, can be viewed as disorders of goal‐directed behavior (Dalley and Robbins 2017; Le Heron et al. 2019; Levy and Dubois 2006). A recent proposal suggests that the mechanisms underlying value‐based decision‐making provide a neurobiologically grounded decision‐making framework within which the co‐occurrence of these problems can be understood (Morris et al. 2022). Broadly, value‐based decision‐making describes the computation and integration of costs and rewards associated with actions toward goals that drive behavior in real‐world settings. Reciprocal connections between the ventral striatum (including the nucleus accumbens) and anterior cingulate cortex (Kolling et al. 2016; Salamone et al. 2007) form a core network within which these processes are instantiated, alongside a broader basal ganglia—medial frontal cortical network (Prevost et al. 2010). Dopamine, noradrenaline, and serotonin are key neuromodulators that encode these processes (Passamonti, Lansdall, and Rowe 2018). Although alterations to this core neural network have been associated with apathy and impulsivity, respectively, in HD, and key neuromodulatory changes have been reported in post‐mortem tissue, the neural regions and neuromodulatory changes in co‐occurring apathy and impulsivity in HD in vivo are yet to be investigated. On a behavioral level, it remains to be teased apart specifically which cognitive processes are disrupted in HD to give rise to either apathy or impulsivity or both. The use of the cognitive framework provided by value‐based decision‐making allows testable hypotheses to be generated, enabling direct investigation of mechanisms at behavioral and physiological levels. These investigations are a clear priority in advancing understanding and therapies for apathy and impulsivity in HD.

Although global apathy and impulsivity scores were correlated in HD, the analysis of their respective subscales suggested it may be specific dimensions of each that drive this relationship. Apathy showed the weakest association with sensation seeking, suggesting sensation seeking does not form part of the behavioral disturbance of co‐occurring apathy and impulsivity in HD but rather captures goal‐directed, adventurous behavior (item examples—“I would enjoy waterskiing,” “I would like to learn to fly an airplane”). Similarly, both social and emotional apathy were not significantly positively correlated with any impulsivity dimensions, a finding previously demonstrated in healthy people (Petitet et al. 2021). Again, this suggests these dimensions do not form part of the core behavioral disruption of co‐occurring apathy and impulsivity but may reflect distinct neurobehavioral changes. We would however suggest some caution in interpreting these changes, given that the neurobiological underpinnings of dimensions of both apathy and impulsivity are not clearly defined (Dickson and Husain 2022; Robbins et al. 2012). Nonetheless, it is clear that not all dimensions of apathy and impulsivity necessarily co‐occur (Petitet et al. 2021). It would seem likely that overlapping dimensions may have more closely linked biological substrates, and future work will need to address this issue, alongside more clearly defining the neurobiology of the dimensions themselves.

The significant association between apathy and reduced wellbeing and poorer quality of life underscores the impact that altered motivation has on everyday life for people with HD. Impulsivity was also correlated with poorer quality of life; however, this seemed to be mainly mediated by its relationship to apathy. Goal‐directed behavior is a process fundamental to survival and wellbeing and enables initiation and continued engagement in activities in home and community settings. Development of efficacious treatments for altered motivation in HD thus may be of tremendous benefit for day‐to‐day functioning. Notwithstanding the lack of such treatments, it remains vital for treating clinicians to assess and monitor these behaviors as well as educate and support both those affected and their caregivers (Anderson et al. 2018).

Although this study was not designed to investigate apathy and impulsivity in the general population, we did include a small group of healthy people, as controls, to aid in interpretation of the HD results. None of the healthy controls in our sample scored above clinical cut‐offs for apathy or impulsivity. On a trait level, however, their behavior was broadly in line with work investigating these issues in people without a brain disorder, which suggests that, even in the absence of pathology, there are intrinsic relationships between these two behavioral types (Petitet et al. 2021). Conceptually, this background relationship is consistent with the co‐occurrence of clinically meaningful apathy and impulsivity, if we assume that the pathological drivers of these behavioral disturbances are impacting a common cognitive system, such as value‐based decision‐making—expression of which can also vary in health.

Assessment of behavioral changes remains challenging. Biological constructs can be quantified (e.g., white blood cell count as a marker of infection), but behaviors are less so and rely either on self‐report or observation (including using behavioral tasks). All questionnaires in our study were self‐reported, so there remains a possibility that a lack of insight into behavior could have skewed responses for some people, although this is less likely an issue at the premanifest to mild disease stages (Isaacs et al. 2020). Anosognosia is common in HD, and patients with it tend to underreport the severity of some behavioral symptoms (Isaacs et al. 2024), which may be a possibility in this study. Despite these challenges, it remains paramount to assess and document behaviors in both clinical and research settings, as they directly affect daily function and caregiver burden (Murley et al. 2021). We did not include people with moderate to severe manifest disease, which means our findings may not be generalizable to later stages in HD, and further work is needed to explore this end of the HD spectrum. A further limitation is that some datapoints were missing for individuals with HD, requiring imputation of missing data for the PCA (not for the raw scale correlations). However, the strength of this study is the use of more than one measure for both apathy and impulsivity, which, when combined using the validated method of principal component analysis, mitigated the effects of missing data while also allowing for more robust detection of these behavioral changes in HD. This is important because of the variability in the use of questionnaires across studies, and additionally because, although widely utilized, not all measures have been validated in the HD population (although some have been used previously in HD). Although our HD sample size was relatively small (*n *= 42), in part because HD is a rare disease, the effect size of the relationship between apathy and impulsivity in other disorders (and seen in the current study) suggests we were adequately powered for the analyses. Nevertheless, repeating this work in an independent HD sample and including both self and informant report scales of apathy and impulsivity will be an important future research goal.

## 5 Conclusion

HD is a complex degenerative disorder that progresses along motor, cognitive, and behavioral axes (McColgan and Tabrizi 2018). The current study builds a foundation for understanding how two crucial elements of this behavioral axis—apathy and impulsivity—relate to each other. This adds weight to a growing evidence base that apathy and impulsivity are linked both as traits and at clinically and functionally disruptive levels. Thus, not only is apathy intrinsically linked to HD, as previously shown, but apathy is also intrinsically linked to impulsivity. This suggests, at a mechanistic level, a common or overlapping neurobiological basis may underpin these behaviors. Future research should focus on elucidating these mechanisms—a vital step toward the development of treatment strategies—with the ultimate goal of meeting an important but unmet treatment need in this population.

## Author Contributions

**Lee‐Anne Morris**: conceptualization, formal analysis, writing–original draft. **Kyla‐Louise Horne**: project administration, resources. **Laura Paermentier**: project administration, resources. **Christina M. Buchanan**: project administration, resources. **Michael MacAskill**: supervision. **Daniel Myall**: resources, software. **Masud Husain**: conceptualization, supervision. **Richard Roxburgh**: project administration, resources. **Tim Anderson**: project administration, resources. **Campbell Le Heron**: conceptualization, formal analysis, writing–review and editing, supervision.

## Conflicts of Interest

The authors declare no conflicts of interest.

### Peer Review

The peer review history for this article is available at https://publons.com/publon/10.1002/brb3.70061.

## Supporting information



Supporting Information

## Data Availability

De‐identified data are available at https://github.com/nzbri/hd‐apathy‐impulsivity.

## Endnotes

1 We did not have neuroimaging data for participants, precluding use of the HD‐Integrated Staging System (Tabrizi et al., 2022).
