# Supplementary material for: Apathy and Impulsivity Co‐Occur in Huntington's Disease
Source: Brain Behav. 2024 Sep 30;14(10):e70061. doi: 10.1002/brb3.70061 (PMC11440026; doi:10.1002/brb3.70061)

Supplementary material

Supplementary Table 1: Huntington's disease - multiple regression model

```
$Models
  Formula
1 "apathy ~ impulsivity_z"
2 "apathy ~ impulsivity_z + bdi_dysphoria_z"
3 "apathy ~ impulsivity_z + bdi_dysphoria_z + moca_z"
4 "apathy ~ impulsivity_z + bdi_dysphoria_z + moca_z + uhdrs_z"
5 "apathy ~ impulsivity_z + bdi_dysphoria_z + moca_z + uhdrs_z + age_z"
6 "apathy ~ impulsivity_z + bdi_dysphoria_z + moca_z + uhdrs_z + age_z + sex"

$Fit.criteria
  Rank Df.res   AIC  AICc   BIC R.squared Adj.R.sq   p.value Shapiro.W Shapiro.p
1    2     40 135.8 136.4 141.0    0.3565   0.3404 2.993e-05    0.9761    0.5170
2    3     39 136.9 138.0 143.8    0.3703   0.3380 1.211e-04    0.9790    0.6241
3    4     38 137.9 139.6 146.6    0.3851   0.3366 3.137e-04    0.9733    0.4239
4    5     37 139.9 142.3 150.3    0.3853   0.3188 1.002e-03    0.9717    0.3766
5    6     36 141.7 145.0 153.9    0.3877   0.3027 2.533e-03    0.9802    0.6691
6    7     35 138.6 143.0 152.5    0.4575   0.3645 9.642e-04    0.9799    0.6587


Winning model: "apathy ~ impulsivity_z"


Call:
lm(formula = apathy ~ impulsivity_z, data = metadata[metadata$group ==
    "HD", ])

Residuals:
    Min      1Q  Median      3Q     Max
-2.8008 -0.7668 -0.1754  0.8820  2.1074

Coefficients:
              Estimate Std. Error t value Pr(>|t|)
(Intercept)     0.2104     0.1809   1.163    0.252
impulsivity_z   0.8131     0.1727   4.707 2.99e-05 ***
---
Signif. codes:  0 ‘***’ 0.001 ‘**’ 0.01 ‘*’ 0.05 ‘.’ 0.1 ‘ ’ 1

Residual standard error: 1.163 on 40 degrees of freedom
Multiple R-squared:  0.3565,	Adjusted R-squared:  0.3404
F-statistic: 22.16 on 1 and 40 DF,  p-value: 2.993e-05
```

Supplementary Figure 1: Apathy and impulsivity in healthy controls

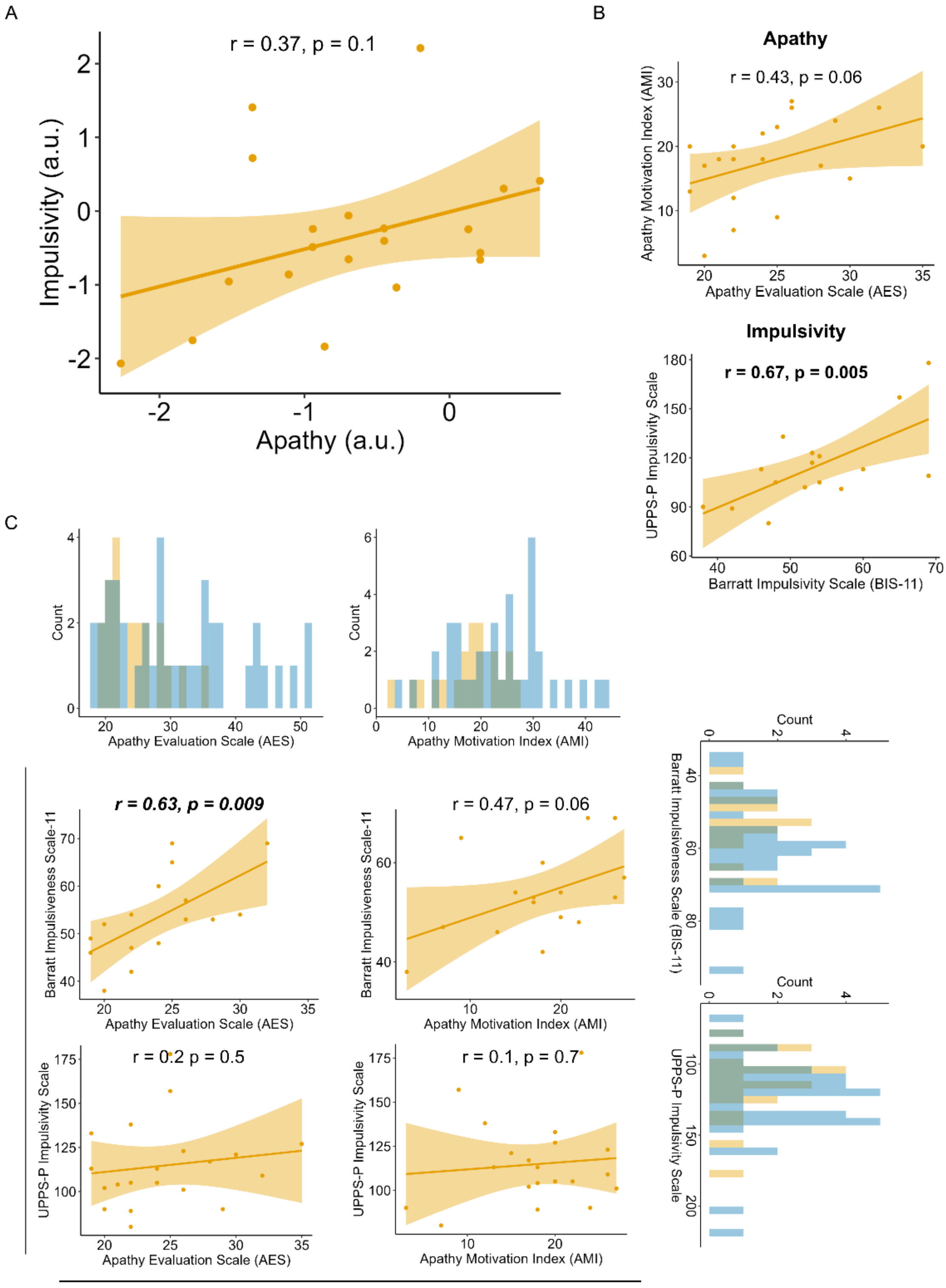

Supplementary Table 3: Healthy controls - multiple regression model

```
$Models
  Formula
1 "apathy ~ impulsivity_z"
2 "apathy ~ impulsivity_z + bdi_dysphoria_z"
3 "apathy ~ impulsivity_z + bdi_dysphoria_z + age_z"
4 "apathy ~ impulsivity_z + bdi_dysphoria_z + age_z + sex"

$Fit.criteria
  Rank Df.res   AIC  AICc   BIC R.squared Adj.R.sq p.value Shapiro.W Shapiro.p
1    2     18 48.09 49.59 51.08    0.1406  0.09288 0.10330    0.9496    0.3604
2    3     17 43.20 45.87 47.18    0.3911  0.31950 0.01474    0.9846    0.9792
3    4     16 44.22 48.50 49.19    0.4204  0.31170 0.02957    0.9822    0.9595
4    5     15 46.06 52.52 52.03    0.4250  0.27160 0.06602    0.9908    0.9989

Winning model: "apathy ~ impulsivity_z + bdi_dysphoria_z"

Call:
lm(formula = apathy ~ impulsivity_z + bdi_dysphoria_z, data = metadata[metadata$g
roup ==
    "Controls", ])

Residuals:
    Min      1Q  Median      3Q     Max 
-1.2061 -0.3276  0.0424  0.3200  1.0769 

Coefficients:
                Estimate Std. Error t value Pr(>|t|)    
(Intercept)      -0.6135     0.1505  -4.076 0.000786 ***
impulsivity_z     0.6022     0.1991   3.025 0.007641 ** 
bdi_dysphoria_z  -0.6637     0.2510  -2.645 0.017028 *  
---
Signif. codes:  0 ‘***’ 0.001 ‘**’ 0.01 ‘*’ 0.05 ‘.’ 0.1 ‘ ’ 1

Residual standard error: 0.6328 on 17 degrees of freedom
Multiple R-squared:  0.3911,	Adjusted R-squared:  0.3195 
F-statistic:  5.46 on 2 and 17 DF,  p-value: 0.01474
```

Supplementary Figure 2: Healthy controls - apathy and impulsivity subscale correlation plot

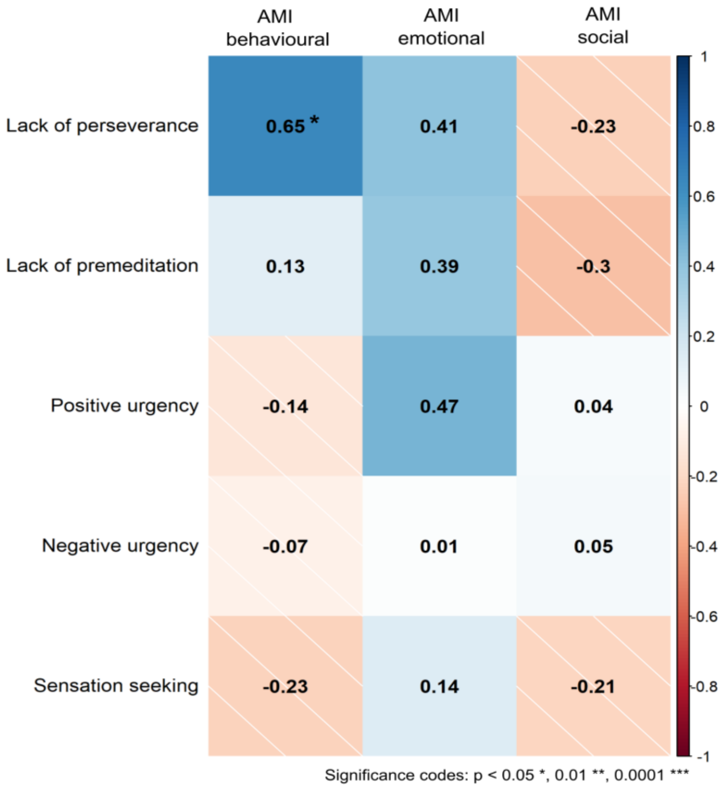

Supplementary Table 4: Healthy controls - wellbeing and quality of life

Controls: wellbeing

```
Call:
lm(formula = who5_percentage_score ~ apathy_z + impulsivity_z +
    bdi_dysphoria_z + age_z + sex, data = metadata[metadata$group ==
    "Controls", ])

Residuals:
    Min      1Q  Median      3Q     Max
-21.753  -3.288   2.524   7.820  19.731

Coefficients:
                Estimate Std. Error t value Pr(>|t|)
(Intercept)      54.2135     5.4768   9.899 1.06e-07 ***
apathy_z          0.5303     7.0348   0.075   0.9410
impulsivity_z   -14.3867     6.6315  -2.169   0.0478 *
bdi_dysphoria_z -12.3425     6.4361  -1.918   0.0758 .
age_z            -4.3181     3.3875  -1.275   0.2232
sexMale          14.8113     7.0432   2.103   0.0540 .
---
Signif. codes:  0 ‘***’ 0.001 ‘**’ 0.01 ‘*’ 0.05 ‘.’ 0.1 ‘ ’ 1

Residual standard error: 13.37 on 14 degrees of freedom
Multiple R-squared:  0.6119,     Adjusted R-squared:  0.4732
F-statistic: 4.414 on 5 and 14 DF,  p-value: 0.0127
```

Controls: quality of life

```
Call:
lm(formula = cantril_score ~ apathy_z + impulsivity_z + +bdi_dysphoria_z +
    age_z + sex, data = metadata[metadata$group == "Controls",
    ])

Residuals:
     Min       1Q   Median       3Q      Max
-2.06013 -0.49469 -0.00435  0.75718  1.60396

Coefficients:
                Estimate Std. Error t value Pr(>|t|)
(Intercept)       7.4384     0.4703  15.816 2.52e-10 ***
apathy_z          0.3801     0.6041   0.629   0.5393
impulsivity_z    -1.0825     0.5695  -1.901   0.0781 .
bdi_dysphoria_z   0.9083     0.5527   1.643   0.1225
age_z             0.2831     0.2909   0.973   0.3470
sexMale           0.4010     0.6048   0.663   0.5181
---
Signif. codes:  0 ‘***’ 0.001 ‘**’ 0.01 ‘*’ 0.05 ‘.’ 0.1 ‘ ’ 1

Residual standard error: 1.148 on 14 degrees of freedom
Multiple R-squared:  0.4038,     Adjusted R-squared:  0.1908
F-statistic: 1.896 on 5 and 14 DF,  p-value: 0.1588
```

Supplementary Figure 3: Healthy controls - wellbeing and quality of life

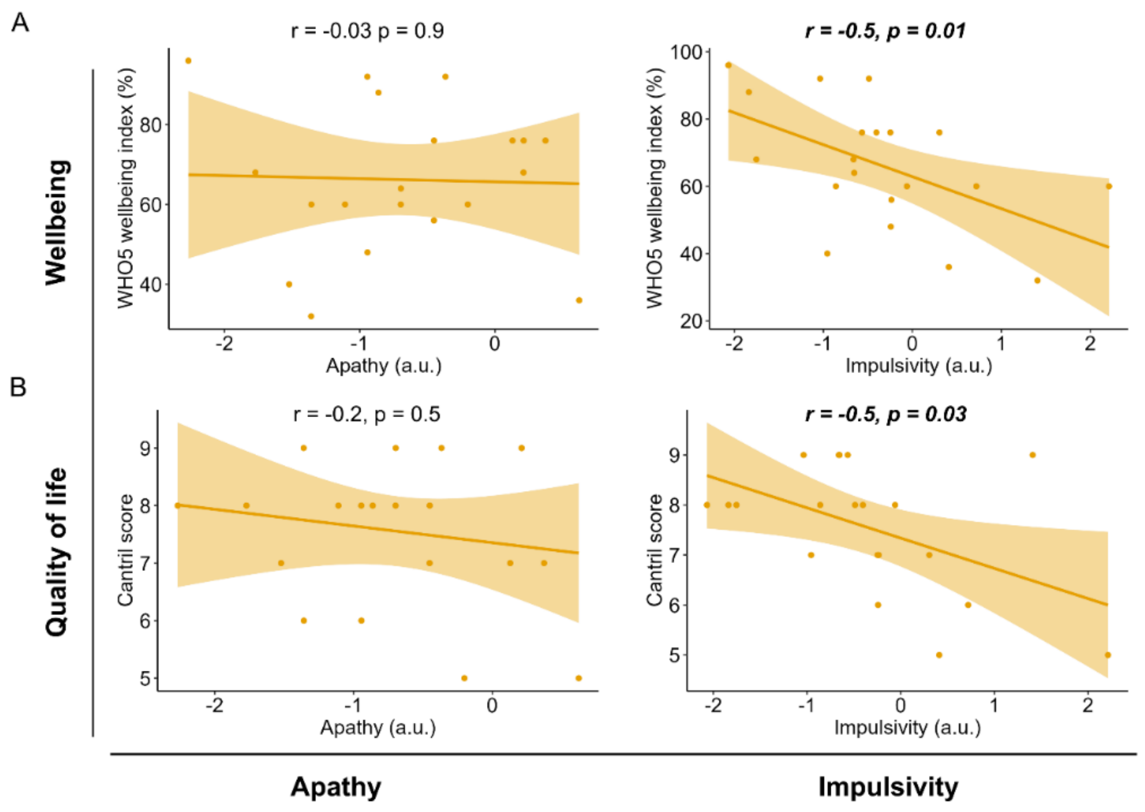

Supplement: Supplementary file 1 — Supporting Information [file BRB3-14-e70061-s001.pdf]
